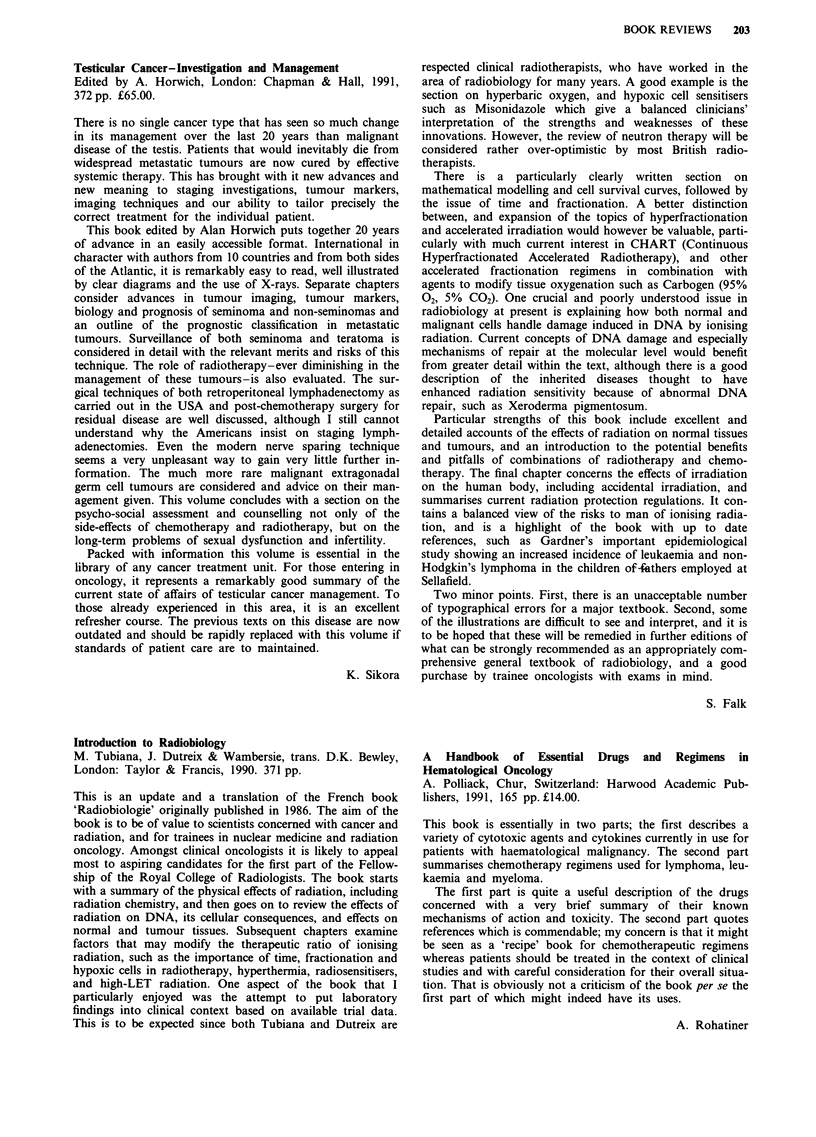# Testicular Cancer-Investigation and Management

**Published:** 1993-01

**Authors:** K. Sikora


**Testicular Cancer – Investigation and Management**
Edited by A. Horwich, London: Chapman & Hall, 1991, 372 pp. £65.00.

There is no single cancer type that has seen so much change in its management over the last 20 years than malignant disease of the testis. Patients that would inevitably die from widespread metastatic tumours are now cured by effective systemic therapy. This has brought with it new advances and new meaning to staging investigations, tumour markers, imaging techniques and our ability to tailor precisely the correct treatment for the individual patient.

This book edited by Alan Horwich puts together 20 years of advance in an easily accessible format. International in character with authors from 10 countries and from both sides of the Atlantic, it is remarkably easy to read, well illustrated by clear diagrams and the use of X-rays. Separate chapters consider advances in tumour imaging, tumour markers, biology and prognosis of seminoma and non-seminomas and an outline of the prognostic classification in metastatic tumours. Surveillance of both seminoma and teratoma is considered in detail with the relevant merits and risks of this technique. The role of radiotherapy – ever diminishing in the management of these tumours – is also evaluated. The surgical techniques of both retroperitoneal lymphadenectomy as carried out in the USA and post-chemotherapy surgery for residual disease are well discussed, although I still cannot understand why the Americans insist on staging lymphadenectomies. Even the modern nerve sparing technique seems a very unpleasant way to gain very little further information. The much more rare malignant extragonadal germ cell tumours are considered and advice on their management given. This volume concludes with a section on the psycho-social assessment and counselling not only of the side-effects of chemotherapy and radiotherapy, but on the long-term problems of sexual dysfunction and infertility.

Packed with information this volume is essential in the library of any cancer treatment unit. For those entering in oncology, it represents a remarkably good summary of the current state of affairs of testicular cancer management. To those already experienced in this area, it is an excellent refresher course. The previous texts on this disease are now outdated and should be rapidly replaced with this volume if standards of patient care are to maintained.

K. Sikora